# Molecular fingerprinting of radiation resistant tumors: Can we apprehend and rehabilitate the suspects?

**DOI:** 10.1186/1471-2407-9-225

**Published:** 2009-07-09

**Authors:** Charles J Rosser, Micah Gaar, Stacy Porvasnik

**Affiliations:** 1 Department of Urology, The University of Florida, Gainesville, Florida, 32610, USA

## Abstract

Radiation therapy continues to be one of the more popular treatment options for localized prostate cancer. One major obstacle to radiation therapy is that there is a limit to the amount of radiation that can be safely delivered to the target organ. Emerging evidence suggests that therapeutic agents targeting specific molecules might be combined with radiation therapy for more effective treatment of tumors. Recent studies suggest that modulation of these molecules by a variety of mechanisms (e.g., gene therapy, antisense oligonucleotides, small interfering RNA) may enhance the efficacy of radiation therapy by modifying the activity of key cell proliferation and survival pathways such as those controlled by Bcl-2, p53, Akt/PTEN and cyclooxygenase-2. In this article, we summarize the findings of recent investigations of radiosensitizing agents in the treatment of prostate cancer.

## Review

Radiotherapy as a single modality has a limited but important role in the overall treatment of solid tumors, specifically prostate cancer. One major difficulty with radiation therapy is that there is a limit to the amount of radiation that can be safely delivered to the target organ [1,2]. For prostate cancer, radiation doses are generally limited to < 80 Gy because of the increased risk of toxicity at higher doses and the lack of clinical evidence that doses > 80 Gy improve local tumor control. Unfortunately, at these dosing levels a significant proportion of tumors are resistant to radiotherapy – either they do not respond to radiotherapy or they recur after treatment. It has become clear that the resistance of human cancers to radiation correlates with the expression of several key genes that regulate different steps of the apoptotic and cell cycle pathway. Thus, the strategy of targeting distinct molecular pathways may translate into higher efficacy, resulting in better survival. Radiation therapy combined with these targeted therapies may exert enhanced antitumor activity through synergic action. The combination treatment could also decrease the toxicity caused by radiation therapy if lower doses could be employed. In this article, we review the current literature on key molecules that have been associated with the development of radiation resistant prostate cancer and discuss novel ways to modulate these molecules to sensitize these tumors to the killing effects of radiation.

In the clinical arena, disease relapse after definitive radiation therapy (i.e., brachytherapy, external beam radiation therapy and proton therapy) is usually identified by an elevated or rising prostate-specific antigen (PSA) profile. Due to the controversy that surrounds identifying disease relapse, a consensus panel was formed to define radiation failure [3]. In addition to the debate about defining failure, controversy exists as to whether this new disease is persistent disease that was inadequately treated or recurrent disease that is refractory to therapy. It is difficult to distinguish persistent disease from recurrent disease. It is possible that persistent disease may have a genotype and phenotype different to recurrent disease since the organ has been exposed to radiation, albeit subtherapeutic doses.

Depending on the initial serum PSA level, Gleason score on biopsy, or clinical stage, 33–85% of patients undergoing external beam radiotherapy (XRT) for localized prostate cancer are biochemically disease-free 5 years after initial radiation therapy [1,2,4-8]. Tumor response to radiation is dependent on the total dose of radiation that can be safely delivered. Prostate tumors are notoriously resistant to total doses of 75 to 80 Gy, but higher doses are not generally administered because of the increased risk of toxicity. Because improved local tumor control is a therapeutic goal for most cancers, various strategies for sensitizing tumors to radiation have been tested over the last 20 years [9-13]. All of these strategies have involved the systemic administration of drugs or other agents that have specific toxicities of their own, which almost always limits pharmacological doses to levels below what is needed to sensitize tumors. The only successful sensitizing strategy thus far has been hormonal deprivation in combination with radiation therapy, which has been demonstrated to improve cause-specific survival in men with advanced prostate cancer [14,15], but still hormonal therapy in this setting is not the panacea. Perhaps molecular targets identified in *in vitro *and *in vivo *work could help improve the efficacy of radiotherapy above and beyond what we see with hormonal therapy. Despite ongoing clinical trials, no sensitizing strategies are currently available for widespread use.

Various genetic abnormalities have been associated with radiation-resistant prostate cancer [16]. Table 1 lists some of the more common genetic abnormalities associated with prostatic disease relapse after definitive radiation therapy [17-25]. The four most common genetic abnormalities reported in the literature, p53, Bcl-2, COX and Akt/PTEN will be reviewed in detail.

**Table 1:** Genes associated with radiation resistant prostate cancer

Major	Mechanism
**Bcl-2**	Mitochondrial membrane protein that blocks the apoptotic death of cells
**P53**	Protein which responds to diverse cellular stresses, regulating cell cycle arrest, apoptosis, senescence, DNA repair, or changes in metabolism
**pAkt**	Protein responsible for cell survival, proliferation, metabolism and angiogenesis
**COX-2**	Enzyme responsible for prostaglandin production which is involved in cellular inflammation and mitogenesis
**Minor**	
**Heat shock proteins 27, 90 **[17-20]	Family of proteins whose expression is increased when cells are exposed to external stresses (e.g., infection, inflammation, hypoxia) and can inhibit apoptosis and activate proteosomes
**Caspase-1 **[21]	Protein which is a member of the cysteine-aspartic acid protease (caspase) family and is central to cell apoptosis as well as inflammation, septic shock, and wound healing
**MDM2 **[22]	Protein with an autoregulatory negative feedback loop p53 effecting cell cycle, apoptosis and tumorigenesis
**Clusterin **[23,24]	Glycoprotein observed to have both pro- and antiapoptotic functions
**Ras **[25]	Family of proteins responsible for signal transduction and cell to cell communication

### PI3K/Akt/PTEN

PI3K is a lipid kinase that can generate phosphatidylinositol-3,4,5-trisphosphate (PI(3,4,5)P3) which has a broad array of functions including inositol phosphate metabolism, phosphatidylinositol signaling system, p53 signaling pathway, cellular proliferation and survival, focal adhesion, and cell-cell communication. PI3K is regulated by its upstream growth factor receptor tyrosine kinases (e.g., EGFR family receptors). In addition, there is cross talk between the PI3K/Akt pathway and mitogen-activated protein kinases (MAP Kinase) pathway, mTOR pathway, and protein kinase A, B and C (PKA, PKB, PKC) pathways [26-29]. Phosphatase and tensin homolog (PTEN), the major negative regulator of the PI3K/Akt pathway, is a tumor suppressor gene that is localized on chromosome l0q23. PTEN is dual specific phosphatase that is involved in controlling cell cycle and apoptosis [30]. The PI3K signaling pathway is frequently aberrantly activated in tumors by mutation or loss of the 3'phospholipid phosphatase. Activation of the PI3K/Akt pathway is associated with three major radioresistance mechanisms: intrinsic radioresistance; tumor-cell proliferation; and hypoxia [31].

**Figure 1:**
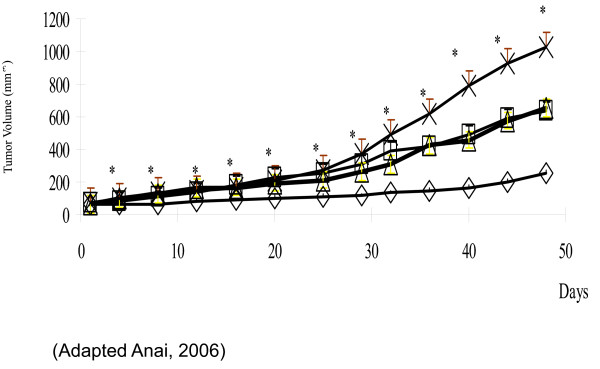
**Effect of PTEN expression and radiation therapy on the growth of human prostate cancer xenograft tumors**. In PC-3 Bcl-2 tumors, a modest, but significant, inhibition of tumor growth was demonstrated in Ad-PTEN alone. Compared to other treatment groups, the addition of irradiation to Ad-PTEN therapy further inhibited tumor growth. mock no XRT, X; mock + XRT, open square; AdPTEN no XRT, open triangle; AdPTEN + XRT, open diamond. (Adapted from Anai, 2006).

Forty-two percent of prostatic tumors have abnormal PTEN/Akt expression [32]. The human prostate cancer cell lines, PC-3, DU-145 and LNCaP are known to be resistant to radiation and thus are widely used in clonogenic radiation assays [33]. One of the cell lines, PC-3, was stably transfected to overexpress Bcl-2 which generated a cell line even more resistant to radiation than its parental lines [34]. Of the above cell lines, only DU-145 has wild-type PTEN and thus suppressed Akt expression (Table 2). Utilizing gene therapy, overexpression of PTEN resulted in down regulation of Akt. PTEN restoration caused significant G1 phase arrest in PC-3 cells. Morphologic changes, most notably apoptotic bodies and reduction in cellular proliferation, were evident in all cell lines. In addition, a reduction in the surviving fractions after 2 Gy of radiation was evident in the majority of cells assayed: PC-3-Bcl-2, reduction from 60.5% to 3.6%; PC-3-Neo, no reduction; LNCaP, reduction from 29.6% to 16.3%; and DU-145, reduction from 32.7% to 25.7%. Thus in select cells, PTEN restoration sensitized these cells to the killing effects of radiation [34]. PTEN restoration was tested in a xenograft model using the human prostate cancer cell line, PC-3-Bcl-2. PTEN expression inhibited xenograft tumor growth, however PTEN expression plus radiation (5 Gy) reduced tumor size to 75% of the untreated tumor (Figure 1). Combination treatment also enhanced apoptosis, inhibited cellular proliferation, and inhibited tumor-induced neovascularity [35].

**Table 2:** Prostate cancer cell lines phenotype

Cell Line	p53 status	Bcl-2 status	PTEN status
**PC-3-Bcl-2**	Mutant	Overexpression	Deleted
**PC-3-Neo**	Mutant	Expression	Deleted
**DU145**	Mutant	Wild type	Wild type
**LNCaP**	Wild type	Overexpression	Deleted

As the next section will illustrate, Bcl-2 expression can result in the development of radiation-resistant cancers. It is interesting that Huang and colleagues demonstrated that PTEN down regulation results in upregulation of Akt and Bcl-2, thus illustrating the possible cross-talk between the Bcl-2 and PI3K/Akt pathway [36].

Unlike for the other molecules we will discuss, limited clinical information is available regarding PI3K/Akt/PTEN conferring radiation resistance in actual human samples. Thus, further studies correlating abnormalities in the PI3K/Akt/PTEN pathway and the development of radiation-resistant disease are needed. If this correlation can be demonstrated clinically, the PI3K/Akt/PTEN pathway may prove to be a desirable therapeutic target that could be used combination with radiation therapy.

### Bcl-2

After p53 the next most commonly discussed genetic aberration associated with radiation resistance is overexpression of Bcl-2. Bcl-2 is the founding member of a protein family composed of regulators of programmed cell death in both normal and abnormal cells. Bcl-2 is a pro-survival multidomain protein that regulates apoptosis by preventing the release of pro-apoptotic factors from mitochondria (e.g., cytochrome c) and subsequent activation of a caspase cascade [37-39]. The role of Bcl-2 as an antiapoptotic molecule is significant in prostate cancer because of the level of tenacity and resistance it grants to a tumor. As a result of these characteristics, it is associated with tumor aggressiveness [40-43].

**Figure 2:**
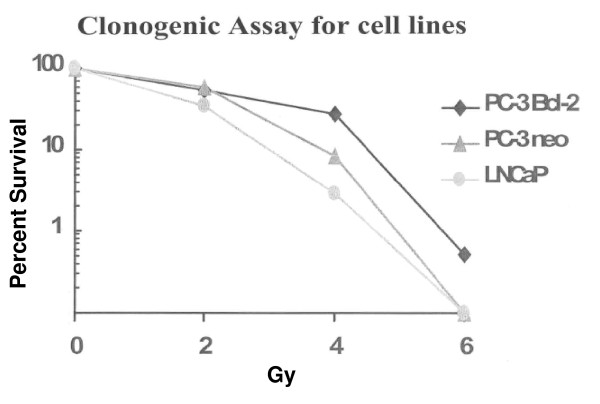
**Clonogenic assay of a panel of human prostate cancer cell lines subjected to irradiation**. Utilizing clonogenic assay, gold standard for monitoring cell survival after irradiation, it was demonstrated that PC-3 cells that overexpressed Bcl-2 were the most radioresistant cells assayed.

**Figure 3:**
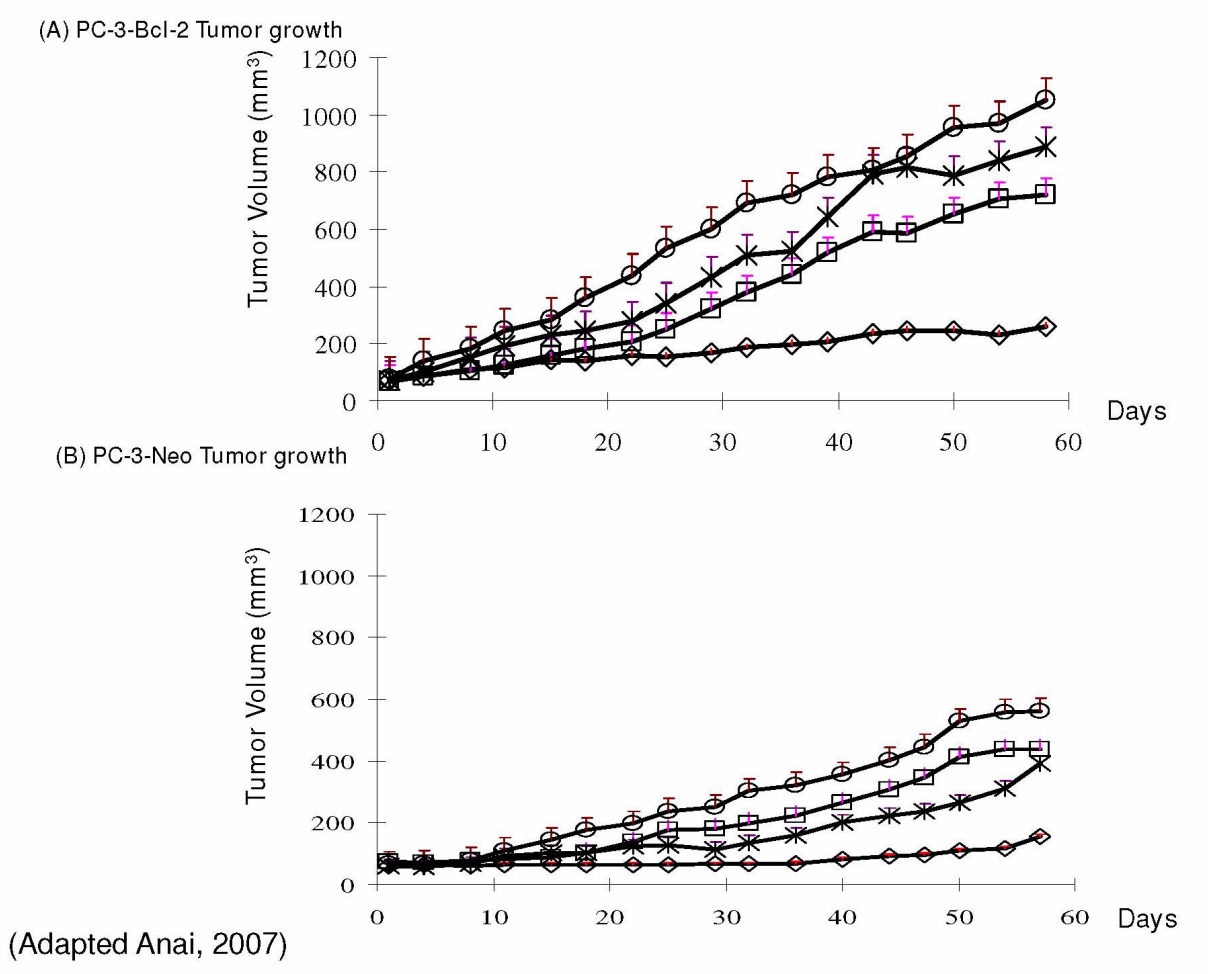
**Effect of Bcl-2 targeted therapy in combination with radiation therapy on the growth of human prostate cancer xenograft tumors**. **(A) **In PC-3 Bcl-2 tumors, growth inhibition was present with antisense Bcl-2 oligonucleotide (ASO) alone or radiation alone. Combining ASO and radiation significantly inhibited of tumor growth compared to other treatment groups. (B) In PC-3-Neo tumors, similar results were evident with combinational therapy resulting in an additive effect with irradiation. mock no XRT, open circle; mock + XRT, X; ASO no XRT, open square; ASO + XRT, open diamond. (Adapted from Anai, 2007).

*In vitro*, exposure of cancer cells to low, non-toxic doses of radiation resulted in the up regulation of Bcl-2, indicating these cells were attempting to adapt to the harmful environment. To confirm that Bcl-2 expression is associated with radiation resistance, human prostate cancer cells PC-3 were stably transfected to express Bcl-2 and subjected to 0–6 Gy radiation. The Bcl-2 expressing clone grew more colonies at higher doses of radiation than the control clones (Figure 2). Utilizing these same cell lines, our group down regulated Bcl-2 expression in this model and studied its effects with and without radiation *in vitro *and *in vivo*. Solely targeting Bcl-2 produced no cytotoxic effects and was associated with G1 cell cycle arrest. The combination of knock-down of Bcl-2 with irradiation sensitized the cells to the killing effects of radiation. In addition, both PC-3-*Bcl-2 *and PC-3-Neo xenografts in mice treated with the combination of targeting Bcl-2 and irradiation were >3 times smaller by volume than were xenografts in mice treated with either modality alone (Figure 3). The combination therapy was also associated with increased rates of apoptosis, decreased rates of angiogenesis, and decreased rates of proliferation [44].

Resistance to radiation therapy in human prostate cancers has been strongly linked to Bcl-2 [45-47]. In a study including 20 men who were radio-naïve and underwent prostatectomy for prostate cancer, no patient in this group was Bcl-2 positive. Among 20 men in the same study who were radio-recurrent and underwent prostatectomy for prostate cancer, 55% of the tumors were Bcl-2 immunopositive (*p *= 0.0004) [48]. Further evidence as to the importance of the expression of key proteins in the Bcl-2 family comes from a prospective, randomized, radiation dose escalation trial that treated 305 men with localized prostate cancer with either 70 or 78 Gy. Tumors were stained using a panel of markers from the Bcl-2 family of proteins including Bcl-2, Bcl-x (antiapoptotic Bcl-2 family member) and Bax (proapoptotic Bcl-2 family member). In the cohort, overexpression of Bcl-2 was observed in 16% of patients; altered Bax expression was observed in 23% of patients; and altered Bcl-x expression was observed in 53% of patients. Kaplan-Meier survival estimates of freedom from biochemical failure (bNED) and the log-rank tests revealed significantly lower rates in association with positive Bcl-2 and altered Bax staining. No correlation was observed between Bcl-x staining; and bNED. Cox proportional hazards multivariate analysis confirmed that Bcl-2 and Bax were independent of pretreatment PSA level, Gleason score and disease stage in predicting bNED [49]. Recently Khor and associates reported on the immunohistochemical results for Bcl-2 in 586 high-risk prostate cancer patients prospectively randomized to the Radiation Therapy Oncology Group (RTOG) trial 92-02 in which XRT was given in conjunction with a short course (STAD) or long course (LTAD) of androgen deprivation therapy. Bcl-2 was positive in 45.6% cases, and Bax expression altered in 53.9% cases. Bcl-2 overexpression was not independently related to biochemical failure, local failure, distant metastasis, cause-specific mortality or overall mortality, although the relative risks (RR) for failure were higher for overall mortality, cause-specific mortality, distant metastasis, and local failure, 1.24 (0.96–1.60), 1.24 (0.79–1.94), 1.37 (0.90–2.08) and 1.27 (0.78–2.07), respectively. In univariate analyses, altered Bax expression was not significantly associated with any of the end points tested either, although there was a trend for treatment failure (RR, 1.30; 95% CI, 0.97–1.73; *p *= 0.0806). However in the multivariate analyses, abnormal Bax expression was significantly associated with overall failure (RR, 1.43; 95% CI, 1.05–1.95; *p *= 0.0226) and marginally with biochemical failure (RR, 1.37; 95% CI, 0.96–1.97; *p *= 0.0851) [50].

Furthermore, the most common practice treatment for patients with locally advanced prostate is radiation therapy in conjunction with androgen deprivation therapy (ADT). ADT treatment triggers an overexpression of Bcl-2 which can lead to androgen independence, a condition associated with advanced prostate cancer [51]. The compelling data presented above illustrates the significance of the Bcl-2 family in conferring radiation resistance in prostatic tumors. Strategies designed to favorably alter this family of survival proteins to enhance the antitumor effect of radiation warrant further clinical evaluation.

### p53

The importance of TP53 (p53) gene as a molecular marker in cancer is demonstrated by the finding that mutations of p53 occur in approximately half of all human malignancies [52]. p53 is a well known protein that acts as a "master watchman" in cell-cycle arrest, programmed cell death, and DNA repair [53-55]. Though some researchers have reported the response to radiation is independent of the cell's p53 status [56], numerous reports are available that demonstrated increased cell kill with irradiation in cells expressing p53 [57].

Gene therapy strategies based on p53 have been shown to reduce tumorigenicity and promote apoptosis in a variety of cell lines and xenograft tumors including colon, head and neck, ovary, and brain [58-66]. Resistance to apoptosis due to cell-cycle disruption has been found to be variably dependent on the existence of p53. p53 is therefore an important protein in the sensitization of prostate cancer to radiation therapy. In an elegant experiment by researchers at MD Anderson Cancer Center, the effects of adenoviral-mediated p53 transgene expression on the radiation response of two human prostate cancer cell lines, LNCaP and PC3 lines, was examined. After correcting for the effect of Ad5-p53 on plating efficiency, the surviving fraction after 2 Gy of radiation was reduced more than 2.5-fold, from 0.187 to 0.072, with transgene p53 expression in the LNCaP cell line. Surviving fraction after 4 Gy was reduced greater than 4.5-fold, from 0.014 to 0.003, after Ad5-p53 treatment. In the PC3 cell line, Ad5-p53 reduced surviving fraction after 2 Gy 1.9-fold from 0.708 to 0.367 and 6-fold at the surviving fraction after 4 Gy from 0.335 to 0.056. Lastly in both cell lines, the combination of Ad5-p53 plus radiation (2 Gy) resulted in supra-additive apoptosis (approximately 20% for LNCaP and approximately 15% for PC3 at 50 MOI) above that seen from the controls [67]. Other groups have been able to corroborate the results of this *in vitro *experiment in human prostate cancer cells [68]. In a follow-up to that study, Cowen presented the *in vivo *results in PC3 and LNCaP xenograft model treated with intratumoral p53 injection and 5 Gy radiation. The time for the PC3 tumors to reach 500 mm^3 ^were calculated as 10.7 days (+/- 0.7) for the saline control, 15.6 days (+/- 1.6) for Ad5-p53, 14.6 days (+/- 1.5) radiation therapy, and 31.4 days (+/- 5.3) for Ad5-p53 plus XRT. Thus, Ad5-p53 plus radiation significantly retarded the growth of xenograft prostate tumors. Furthermore in LNCaP xenograft tumors where serum PSA levels were used to judge treatment efficacy, treatment with Ad5-p53 plus 5 Gy resulted in significantly fewer PSA failures (<30%), as compared with failures with Ad5-p53 alone (64–73%) and the other controls (approximately 80–100%) [69]. These studies indicate that prostatic tumor growth could be inhibited with the over expression of p53 in combination with radiation therapy.

Several small, retrospective studies in men with prostate cancer have suggested that abnormal p53 expression is associated with poor outcomes [70-74]. In addition, prospective clinical trials have clearly demonstrated the prognostic implications of abnormally expressed p53 in men treated with XRT. For example, tissue samples from 777 men enrolled in RTOG 92-02, the seminal study that compared STAD with XRT to LTAD + XRT, with locally-advanced prostate cancer were analyzed for p53 expression. Abnormal p53 expression was defined as 20% or more tumor cells with positive nuclei. Abnormal p53 was detected in 168 (21.6%) of 777 cases and was significantly associated with cause-specific mortality (adjusted hazard ratio [HR] = 1.89; 95% confidence interval [CI] 1.14 – 3.14; *p *= 0.014) and distant metastasis (adjusted HR = 1.72; 95% CI 1.13–2.62; *p *= 0.013). When patients were divided into subgroups according to assigned treatment, only the subgroup of patients who underwent STAD + RT showed significant correlation between p53 status and cause-specific mortality (adjusted HR = 2.43; 95% CI = 1.32–4.49; *p *= 0.0044). This increase in cause-specific mortality could be reduced with LTAD [73]. Similarly, in RTOG 86-10, a phase III trial of Zoladex and flutamide in locally advanced carcinoma of the prostate treated with definitive radiotherapy, patients were assessed for the prognostic significance of abnormal p53 expression. One hundred twenty-nine (27%) of the 471 patients entered in the trial had sufficient tumor material for analysis. Abnormal p53 protein expression was detected in the tumors of 23 (18%) of these 129 patients. Statistically significant associations were found on a multivariate analysis between the presence of abnormal p53 protein expression and increased incidence of distant metastases, decreased progression-free survival, and decreased overall survival. No association was found between abnormal p53 protein expression and the time to local progression [75].

In addition, D'Amico reported the results of CALGB 9682 in which 180 men with clinical stage T1c-T3cN0M0 adenocarcinoma of the prostate. They were treated with ADT and assessed with endorectal magnetic resonance imaging (eMRI) for change in tumor volume (TV) and associated PSA outcome. Of these subjects, 141 had sufficient tissue to assess p53 expression. After a median follow-up of 6.9 years and adjusting for PSA level, Gleason score, clinical stage, and change in tumor volume, men with abnormal p53 expression compared with normal were at increased risk of PSA failure (hazard ratio [HR]: 2.8; 95% confidence interval [CI]) [76].

Knowing the significance of p53 expression, the next hurdle is whether we can manipulate p53 expression to obtain more favorably outcomes. Researchers studying other tumor types have published on the success of this approach [77]. For prostate cancer, only one significant trial stands out. In a phase I/II clinical trial, investigators at MD Anderson Cancer Center, administered intraprostatic injections of Ad-p53 on three separate occasions prior to radical prostatectomy in subjects with high-risk localized prostate cancer. There were no grade 3 or 4 adverse events related to p53 administration. Of the 11 patients with negative baseline immunostaining for p53 protein, 10 had positive p53 immunostaining after the administration of p53, and 8 had an increase in apoptotic cells [78]. Though adenoviral gene therapy has its limitations, this study is an excellent example of proof of the concept that p53 expression may restore the cell's normal function which can then assist in the eradication of the tumor. Further research is needed to explore targeting p53 in conjunction with radiation therapy in the treatment of prostate cancer.

### COX-2

Cyclooxygenase is a family of isozymes that convert arachidonic acid to prostaglandins and other eicosanoids. COX-1, ubiquitously expressed in almost all tissue, is important for the maintenance of homeostatic function [79]. COX-2 has been well-characterized as a key component in the inflammatory pathway of such disorders as rheumatoid arthritis and Parkinson's Disease [80,81]. In addition, COX-2 is overexpressed in 80% of cancers of the breast, colon, esophagus, liver, lung, pancreas, cervix, head and neck, and prostate [82-85]. COX-2 is induced by growth factors, tumor promoters, and cytokines and is subject to transcriptional and translational regulation and degradation [86]. Thus, COX-2 is another important cell survival factor. Overexpression of COX-2 or its related products confers resistance to cells undergoing chemotherapy or radiation therapy induced apoptosis.

Similar to what was reported for Bcl-2, cancer cells exposed to low doses of radiation up-regulated COX-2 expression as a possible means to survive the radiation exposure [87]. As described above, therapeutic gamma radiation exerts its effect on the function and/or survival of cells by interacting with biologically important molecules, either directly or indirectly. Exposure to radiation can directly affect DNA structure, which can in turn influence the subtle balance of the expression of genes whose products are involved in promoting cell survival or triggering cell death. Interestingly, other environmental stressors, including hypoxia and acidosis, resulted in an up-regulation of COX-2 expression [87].

In a series of *in vitro *clonogenic assays, the human prostate cancer cell line LNCaP was stably transfected to overexpress COX-2. LNCaP-COX-2 cells were significantly more resistant to radiation therapy compared to LNCaP-Neo cells. However, the addition of celecoxib, a selective COX-2 inhibitor that blocks the production of prostaglandins primarily via inhibition of cyclooxygenase-2, sensitized LNCaP-COX-2 cells to the cytocidal effects of radiation. Moreover, we confirmed that COX-2 overexpression was associated with overexpression of pAkt and CA IX, two key genes associated with poor tumor response to radiation [87]. Other preclinical reports have confirmed that COX-2 overexpression and/or subsequently targeting COX-2 with these COX-2 inhibitors may render cells susceptible to the killing effects of the radiation [88-91].

Although limited *in vivo *data are available as to the significance of COX-2 overexpression as related to its ability to confer radiation resistance, recently Khor and colleagues reported the immunohistochemical results for COX-2 in 586 patients enrolled in Radiation Therapy Oncology Group (RTOG) 92-02. As previously stated, in the RTOG 92-02 trial patients were randomly assigned to treatment with STAD plus radiotherapy or LTAD plus radiotherapy. In multivariate analyses, the intensity of COX-2 staining as a continuous covariate was an independent predictor of distant metastasis (hazard ratio [HR] 1.181; 95% CI 1.077–1.295); biochemical failure (HR 1.073 [1.014–1.134]); and any failure (HR 1.068 [1.015–1.124]) [92].

Both the Bcl-2 pathway and COX-2 pathway overlap at phosphatidylinositol 3-kinase (PI3K)/Akt, making these molecules attractive targets. The above data support a pivotal role for COX-2 expression in tumors treated with irradiation and thus justify studies to evaluate the clinical application of targeting COX-2 in patients undergoing radiation therapy as a treatment for prostate cancer.

### Future Directions

The expression of the aforementioned genes has been reliably linked to the development of radiation-resistance cancers. It is feasible to conclude that a litany of other survival genes may be associated with the development of radiation resistant cancers. Limited data are available from the use of high throughput technology (e.g., genomic and proteomic) in the discovery of new biomarkers of which influence resistance to radiation in prostate tumors. As is clearly evident here, no single molecule is responsible for the development and growth of radiation-resistance prostate cancer. As is common in the development of primary tumors, multiple genes are aberrant. The importance of the redundancy in the aberrant genes is that they are likely to ensure the survival of tumors. The ability to target more than one gene is an attractive approach. The recent flourish in the clinical development of more than 30 targeted protein kinase inhibitors designed to inhibit angiogenesis, tumor growth and progression attests to this [93,94]. Specifically, Imatinib mesylate [95], Sorafenib [96], Sunitinib malate [97], and Temsirolimus [98] have demonstrated efficiency in solid tumors including renal cell carcinoma. Due to the multiple targets inhibited by these multikinase inhibitors, effects in different tumor types are likely to be mediated through a variety of mechanisms. Limited forays combining these inhibitors with radiation therapy in a variety of tumor types have produced encouraging results [99]. We clearly recognize the roadblocks in designing clinical trials to test new agents for radiosensitizing since it is difficult to obtain tissue after radiation therapy for prostate cancer. However, the question begs consideration, can targeted therapy effectively treat these cancers? Similar to what we have seen in other cancers (e.g., testis, lymphoma, colon), optimal therapy will consist of a therapeutic 'cocktail' involving traditional chemotherapy and the new targeted therapies in conjunction with radiation therapy, which has also evolved over the past decade.

The importance of incorporating these targeted therapies may be similar to incorporating ADT with radiation therapy. Preclinical studies have demonstrated that ADT can induce apoptosis and inhibit angiogenesis [100]; outcomes that may be potentitated by radiation therapy. These preclinical studies subsequently were translated into a clinical therapeutic advantage in men with high risk localized prostate cancer. Only four targeted therapeutic trials are currently open (Table 3) in men with high risk prostate cancer treated with radiation therapy. A more concerted attempt must be made to bring promising targeted therapeutics to clinical trial to determine treatment efficacy.

**Table 3:** Accruing Clinical Trials Combining Targeted Therapy with radiation Therapy

Agent	Institute
Sunitinib	MD Anderson Cancer Center
SU5416	University of Chicago
Bevacizumab	1) Benaroya Research Institute 2) Virginia Mason
RAD001	Shelba Medical Center

## Conclusion

Radiation therapy continues to be one of the more popular treatment options for localized prostate cancer. However, recent studies suggest that abnormal expression of Bcl-2, p53, Akt/PTEN and cyclooxygenase-2 may render tumors resistant to the killing effects of radiation. However modulation of these molecules by a variety of mechanisms (e.g., gene therapy, antisense oligonucleotides, small interfering RNA) may enhance the efficacy of radiation therapy by modifying the activity of these key cell proliferation and survival pathways.

## List of abbreviations

MOI: Multiplicity Of Infection; Ad: adenovirus; PTEN: Phosphatase and tensin; PI3K: Phosphatidylinositol 3 kinase; MAP kinase: mitogen-activated protein kinases; PKA, PKB, PKC: protein kinase A, B and C; RTOG: Radiation Therapy Oncology Group; STAD: short course of androgen deprivation therapy; LTAD: long course of androgen deprivation therapy; eMRI: endorectal magnetic resonance imaging (eMRI); TV: tumor volume; Gy: Gray; PSA: prostate specific antigen; XRT: radiation therapy; bNED: biochemical no evidence of disease; ADT: androgen deprivation therapy; HR: hazard rates; CI: confidence interval.

## Competing interests

The authors declare that they have no competing interests.

## Authors' contributions

MG collected data and drafted the manuscript. SP assisted in collecting data and revising the manuscript. CJR conceived the project, and participated in its design and coordination. All authors read and approved the final manuscript.

## Pre-publication history

The pre-publication history for this paper can be accessed here:

http://www.biomedcentral.com/1471-2407/9/225/prepub
